# Mental Health in German Paralympic Athletes During the 1st Year of the COVID-19 Pandemic Compared to a General Population Sample

**DOI:** 10.3389/fspor.2022.870692

**Published:** 2022-04-14

**Authors:** Aglaja Busch, Eva Johanna Kubosch, Antonia Bendau, Rainer Leonhart, Verena Meidl, Berit Bretthauer, Moritz Bruno Petzold, Petra Dallmann, Nina Wrobel, Jens Plag, Andreas Ströhle, Anja Hirschmüller

**Affiliations:** ^1^Outpatient Clinic, Sport Medicine and Sports Orthopedics, University of Potsdam, Potsdam, Germany; ^2^Division Physiotherapy, Department of Health Professions, Bern University of Applied Sciences, Bern, Switzerland; ^3^Department of Orthopedics and Trauma Surgery, Medical Center—Albert-Ludwigs-University of Freiburg, Freiburg, Germany; ^4^Department of Psychiatry and Neurosciences CCM, Charité—Universitätsmedizin Berlin, Freie Universität Berlin and Humboldt Universität zu Berlin, Berlin, Germany; ^5^Department of Psychology, Albert-Ludwigs-University of Freiburg, Freiburg, Germany; ^6^Department of General Psychiatry, Centre for Psychosocial Medicine, University of Heidelberg, Heidelberg, Germany; ^7^Department of Internal Medicine, Medical Center—Albert-Ludwigs-University of Freiburg, Freiburg, Germany; ^8^ALTIUS Swiss Sportmed Center AG, Rheinfelden, Switzerland

**Keywords:** paralympic sport, elite athlete, PHQ-4, depression, anxiety, SARS-CoV-2

## Abstract

**Introduction:**

The COVID-19 pandemic has huge influences on daily life and is not only associated with physical but also with major psychological impacts. Mental health problems and disorders are frequently present in elite paralympic athletes. Due to the pandemic situation, new stressors (e.g., loss of routine, financial insecurity) might act upon the athletes. Therefore, the assessment of mental health in athletes during the COVID-19 pandemic is important to identify prevalence of psychological problems and propose countermeasures.

**Methods:**

The mental health of German paralympic athletes was longitudinally monitored (starting in May 2019). The athletes completed the Patient Health Questionnaire 4 (PHQ-4) on a weekly basis and reported a stress level, training hours, and training load. During the pandemic, 8 measurement time points (March 2020 to April 2021) were used to reflect the psychological health course of the athletes. In parallel, a convenience sample of the general population was questioned about their psychological distress, including the PHQ-4. To be included in the analysis, participants of both groups had to complete at least 4 measurement time points. Matching of the para-athletes and the general population sample was prioritized upon completion of the same measurement time points, gender, and age.

**Results:**

Seventy-eight paralympic athletes (40 women, 38 men, age: 29.8 ± 11.4 years) met the inclusion criteria. Seventy-eight matched pairs of the general population (40 women; 38 men; age: 30.5 ± 10.9 years) were identified. The para-athletes had a significantly (*p* <0.0001; 0.39 < *r* <0.48) lower PHQ-4 value at each measurement time point compared to the matched control group. No significant age or sex differences were evident regarding the symptom burden. In para-athletes, no significant and a weak positive correlation was found between decreased training load and PHQ-4 values and a stress level, respectively. Reduced physical activity was significantly (*p* <0.0001) associated with higher PHQ-4 values in the general population sample.

**Discussion:**

Lower PHQ-4 values were reported by the para-athletes compared to the general population sample. However, small sample sizes must be considered while interpreting the data. Nevertheless, adequate support for individuals suffering from severe psychopathological symptoms should be provided for para-athletes as well as for the general population.

## Introduction

The infectious coronavirus disease 19 (COVID-19) caused many severe illnesses and deaths worldwide and resulted in a global alert situation with major restrictions of the daily life. This had impacts on an individual level and close relationships (e.g., social distancing and isolation) as well as on expanded networks and the societal level (e.g., postponement or cancelations of major events). These changes were probably not only affecting the physical health but could also influence psychological health (Wu et al., 2021). A global multicenter study has shown a negative effect of home confinement on emotional states (Ammar et al., 2020). Reviews have shown pooled prevalence of 16–46% of depressive symptoms and 22–37% of anxiety disorders in the global population during the COVID-19 pandemic (Chen et al., 2021; Pappa et al., 2021). This prevalence is higher than one would expect in non-pandemic times and highlights the importance of a comprehensive understanding of the mental health status during the pandemic.

Ramifications of the COVID-19 pandemic were impacting people in all population groups, including elite para-athletes. In general, mental health symptoms and disorders are frequent in elite athletes (Reardon et al., 2019; Henriksen et al., 2020). The loss of daily routines, new goal setting as major sport events were postponed or canceled, and financial insecurity could lead to psychological distress. These changes can be considered as non-normative transitions (i.e., injury or de-selection) and are allied with feelings of depression and anxiety (Knowles et al., 2021). Reviews on mental health in athletes reported high prevalence of psychological health issues with depression, tension, and anxiety as the most rated emotions (Haan et al., 2021; Jurecka et al., 2021). However, mainly cross-sectional observation and few longitudinal studies were presented, lacking the inclusion of athletes with disabilities.

Limited literature is available, investigating the impact of COVID-19 on training and mental health in elite para-athletes. One study showed that training time was significantly reduced, and the majority of para-athletes reported insufficient contact with coaches and assistants (Urbański et al., 2021). Contrarily, training volume and fitness were not affected in para-cyclists and para-triathletes (Shaw et al., 2021). Focusing on mental health, one study on para-swimmers showed decreased apathy in female swimmers compared to their able-bodied pairs during state of emergency. This was elevating again after restrictions were eased (Kaneda et al., 2021). Furthermore, para-athletes expressed their concerns about their health and their social contacts during the pandemic situation. Nearly half of the participating para-athletes opine that people with disabilities are affected differently during the COVID-19 pandemic compared to able-bodied persons (Kubosch et al., 2021).

The assessment of the mental health in para-athletes over the course of the pandemic situation is important to identify prevalence of psychological problems and propose countermeasures. Therefore, the purpose of this longitudinal study was to investigate the mental health in paralympic athletes in comparison to a matched sample of the general population. It was hypothesized that the para-athletes show higher mental health distress than the general population sample.

## Materials and Methods

### Study Design and Participants

Data in this longitudinal observation study were assessed at eight time points during the 1st year of the COVID-19 pandemic (March 2020 – April 2021). These time points were: March 27 to April 6, 2020 (T1), April 24 to May 4, 2020 (T2), May 15 to May 25, 2020 (T3), June 5 to June 15, 2020 (T4), September 25 to October 5, 2020 (T5), October 23 to November 2, 2020 (T6), January 1 to January 11, 2021 (T7), and March 26 to April 5, 2021 (T8). Details of the pandemic and training/sport situation at each of the eight measurement periods are displayed in Table 1.

**Table 1:** Measurement time points (T1-8) and the according pandemic situation in Germany (information of countermeasures was based on the following information: Presse- und Informationsamt der Bundesregierung).

**Time point**	**Pandemic situation**	**Training & sport situation**
T1 (March 27 – April 6 2020)	Lockdown	No organized indoor & outdoor sport and postponement of the Tokyo 2020 Games
T2 (April 24 – May 4 2020)	Stepwise reduction of restrictive measures	Outdoor individual/contact less recreational and high-performance sport stepwise possible
T3 (May 15 – May 25 2020)	Reopening conditions with few restrictive measures	Outdoor individual/contactless recreational and training high-performance sport stepwise possible
T4 (June 5 – June 15 2020)	First obligations for face masks and restrictions in regions with 50 new cases per 10.000 inhabitants in the last 7 days	Contactless recreational and normal training in high performance sport possible
T5 (September 25 – October 5, 2020)	No further easing of the restrictions and restrictions in regions with above 50 new cases per 10.000 inhabitants in the last 7 days	Contactless recreational and training in small groups in high performance sport possible
T6 (October 23 – November 2 2020)	Semi-lockdown	No recreational sports except for individual sport outside
T7 (January 1 – January 11 2021)	Second lockdown; Start of the vaccination for most vulnerable persons of the population	No recreational sports except for individual sport outside
T8 (March 26 to April 5 2021)	Ongoing strict restrictions; Ongoing vaccination offer but with restrictions for the general population, also for para-athletes	No recreational sports except for individual sport outside

The participants of this study were obtained from two different samples as described in more detail below. All the participants gave written informed consent prior to participation. Only the participants that completed at least four assessment periods had been included in the matching process and further analysis. Matching was completed upon three priorities in descending order: the same completed measurement time points, sex, and age. The paralympic sample included the participants starting from age 16 and the general population sample from age 18. Therefore, the general population sample is slightly older than the paralympic sample.

#### Paralympic Sample

All paralympic athletes aiming for participating in the Paralympic Summer or Winter Games were invited to take part in longitudinal monitoring of physical and mental health problems being launched in May 2019 (Busch et al., 2019, 2021). In total, 120 athletes participated in the longitudinal monitoring at any given time point of the observation period, of which 78 completed the questionnaire at a minimum of four assessment time points and were finally included in the present study. Assessments were provided *via* a web application on the platform Athlete Monitoring (FitStats Technologies Inc., Moncton, Canada). The project followed the declaration of Helsinki and was approved by the institutional ethics committee (245/18), and it was registered on drks.de (DRKS00015771).

#### General Population Sample

The reference sample stems from a non-probability convenience sample of the general population in Germany (Bendau et al., 2021). With a longitudinally online survey on the platform SoSci-Survey (SoSciSurvery GmbH, Munich, Germany), a total of 8,041 adults were assessed repeatedly at up to eight waves during the 1st year of the COVID-19 pandemic. Recruitment took part *via* news portals and the homepage as well as social media channels (Twitter, Instagram, Facebook) of the Charité – Universitätsmedizin Berlin. The ethics committee of the Charité – Universitätsmedizin Berlin approved the study (EA1/071/20), and it was registered on clinicaltrials.gov (NCT04331106).

### Assessments

Demographic variables such as gender and age were assessed for both sample cohort participants at the beginning of their respective study participation.

The patient health questionnaire 4(PHQ-4) (Kroenke et al., 2009) was used to assess mental health in the participants. The questionnaire consisted of a 2-item depression scale (PHQ-2) and a 2-item anxiety scale (GAD-2), asking for the frequency of impairments due to psychological complaints in the past 2 weeks. Points are awarded for the respective frequency (0 to 3 points), and an overall score is calculated (max. 12 points; higher values indicate more burden) (Kroenke et al., 2009). Additionally, the subjective stress level on a scale from 0 (no stress) to 10 (maximum stress) was assessed in all paralympic athletes.

Furthermore, the subjective training load was rated by the para-athletes. The athletes responded in a 4-point Likert scale (Likert, 1932), ranging from 1 (very low/rest) to 4 (higher than normal). In addition, the training minutes per week were obtained from all para-athletes. Contrarily, the general population sample was given the statement to have been less physically active since the beginning of the COVID-19 pandemic. The responses were scaled in a 6-point Likert scale, ranging from 1 (does not apply at all) to 6 (does apply fully).

### Statistical Analysis

Identified matched pairs were entered into one spreadsheet for further analysis. Descriptive statistics were presented in percentage (%), mean (M), standard deviation (SD), and inter quartile range (IQR). For comparison of the PHQ-4 sum score between the two cohorts at each measurement time point, a Mann–Whitney *U*-test (α = 0.05), and effect size calculation was performed using the statistical software R (R, version 4.0). Effect sizes were defined as small (0.1), medium (0.3), and strong (0.5) (Cohen, 1988). For analysis of the PHQ-4 sum score over all eight measurement time points, a latent growth model with linear term was obtained. In a latent growth model, individual- and group-level analyses are combined, allowing for identification of appropriate modeling of the growth curve and the importance of variables on the rate of development (Duncan and Duncan, 2004). In the present analysis, an examination whether a linear trend is evident and whether the variables sample membership (para-athlete or general population), age, or sex influence the PHQ4-sum score was performed. MPlus 8.7 was used for this analysis.

Furthermore, spearman rank correlations between the PHQ-4 sum score, stress level, and the obtained subjective training load and training minutes per week at assessment time points of the para-athletes were calculated (R, version 4.0). For the matched sample, the spearman rank correlation between the PHQ-4 sum score and the subjective activity status was performed. Correlation coefficients were defined as weak (0.1–0.3), moderate (0.4–0.6), strong (0.7–0.9), and perfect (>0.9) according to Dancey and Reidy (Akoglu, 2018).

## Results

Seventy-eight paralympic athletes (40 women, 38 men, age: 29.8 ± 11.4 years) met the inclusion criteria. Thus, 78 matched pairs of the general population sample (40 women; 38 men; age, 30.5 ± 10.9 years) were identified. Forty-two participants completed all eight measurement time points; seven participants, seven; three participants, six; two participants, five, and 27 participants filled out four measurement time points. In five cases, one and in two cases, two measurement time points were missing in the general population sample compared to their para-athlete pairs.

Descriptive statistics of the PHQ-4 sum score of both cohorts and an additional stress level of the para-athletes are presented in Table 2 (details of the distribution per cohort and measurement time point can be found in Supplementary 1). PHQ-4 sum scores were significantly lower (*p* <0.0001) in the paralympic cohort at each measurement time point compared to the matched cohort with a medium to strong effect size (0.39 < *r* <0.48). In general, the average PHQ-4 sum score displayed little change over the course of the assessments in both samples. Furthermore, over all eight measurement time points, a latent growth model with acceptable fit was found using maximum likelihood estimation. The following fit parameters were shown for the model: Akaike Information Criterion (AIC) = 4053.857, Bayesian Information Criterion (BIC) = 4111.804, Chi-Square Test of Model with 49 degrees of freedom = 78.890 (*p* = 0.0043). Root Mean Square Error of Approximation (RMSEA) was acceptable at.063 (90% confidence interval: 0.035–0.087, probability of RMSEA ≤ 0.05 = 0.201). The comparative fit index (CFI = 0.969) and the Tucker–Lewis index (TLI = 0.968) demonstrate a very good fit of the model. On the intercept, being part of the paralympic team had a significant effect on the PHQ-4 value (*r* = −0.477, *p* <0.001), but no statistically significant effect of sex or age was found. There was no significant influence by the three entered variables on the slope of the model. Models *R*^2^ values showed that at least 74% of the PHQ-4 value variance at each time point can be explained by the group membership (para-athlete vs. general population) (cf. Table 3).

**Table 2:** PHQ-4 sum scores, stress levels, and training (minutes per week) per group and measurement time points (T1-8).

		**T1**	**T2**	**T3**	**T4**	**T5**	**T6**	**T7**	**T8**
Para- athlete sample	PHQ-4 sum-score [0–12; M ± SD (IQR)]	1 ± 1.6 (1.5)	0.9 ± 1.5 (2)	1.2 ± 2 (2)	0.9 ± 1.6 (1)	1.2 ± 1.9 (2)	1.6 ± 2.6 (3)	1.1 ± 2.1 (2)	1.3 ± 2.1 (2)
	Stress level [0–10; M ± SD (IQR)]	2.8 ± 2.3 (3)	3.1 ± 2.3 (4)	3.1 ± 2.6 (4)	3.2 ± 2.3 (3.5)	3.6 ± 3.6 (3)	3.7 ± 3.7 (3)	2.7 ± 2.7 (3)	3.9 ± 2.4 (3.8)
	Training (minutes/week; M ± SD)	491 ± 380	542 ± 392	611 ± 435	679 ± 497	626 ± 425	655 ± 405	508 ± 402	703 ± 411
General population sample	PHQ-4 sum-score [0–12; M ± SD (IQR)]	3.7 ± 3 (4.5)	3.9 ± 2.9 (3)	4 ± 3 (4)	3.4 ± 3.1 (4)	3.8 ± 3.5 (5)	3.5 ± 3.2 (4)	3.8 ± 3.8 (5)	4 ± 3.3 (5)

*M, Mean; SD, standard deviation; IQR, interquartile range*.

**Table 3:** Observed and latent *R*^2^ of PHQ-4 values at each measurement time point (T1-8).

**Observed**	**Two-tailed**			
**Variable**	**Estimate**	**S.E**.	**Est./S.E**.	***P*-value**
T1	0.787	0.041	19.302	<0.0001
T2	0.809	0.035	22.892	<0.0001
T3	0.797	0.035	22.967	<0.0001
T4	0.779	0.035	22.233	<0.0001
T5	0.741	0.035	2.955	<0.0001
T6	0.729	0.036	2.054	<0.0001
T7	0.823	0.032	25.673	<0.0001
T8	0.760	0.037	2.725	<0.0001
**Latent**			
Intercept	0.231	0.070	3.298	0.001
Slope	0.034	0.050	0.682	0.495

*S.E., standard error; Est./S.E., estimated standard error*.

**Figure 1:**
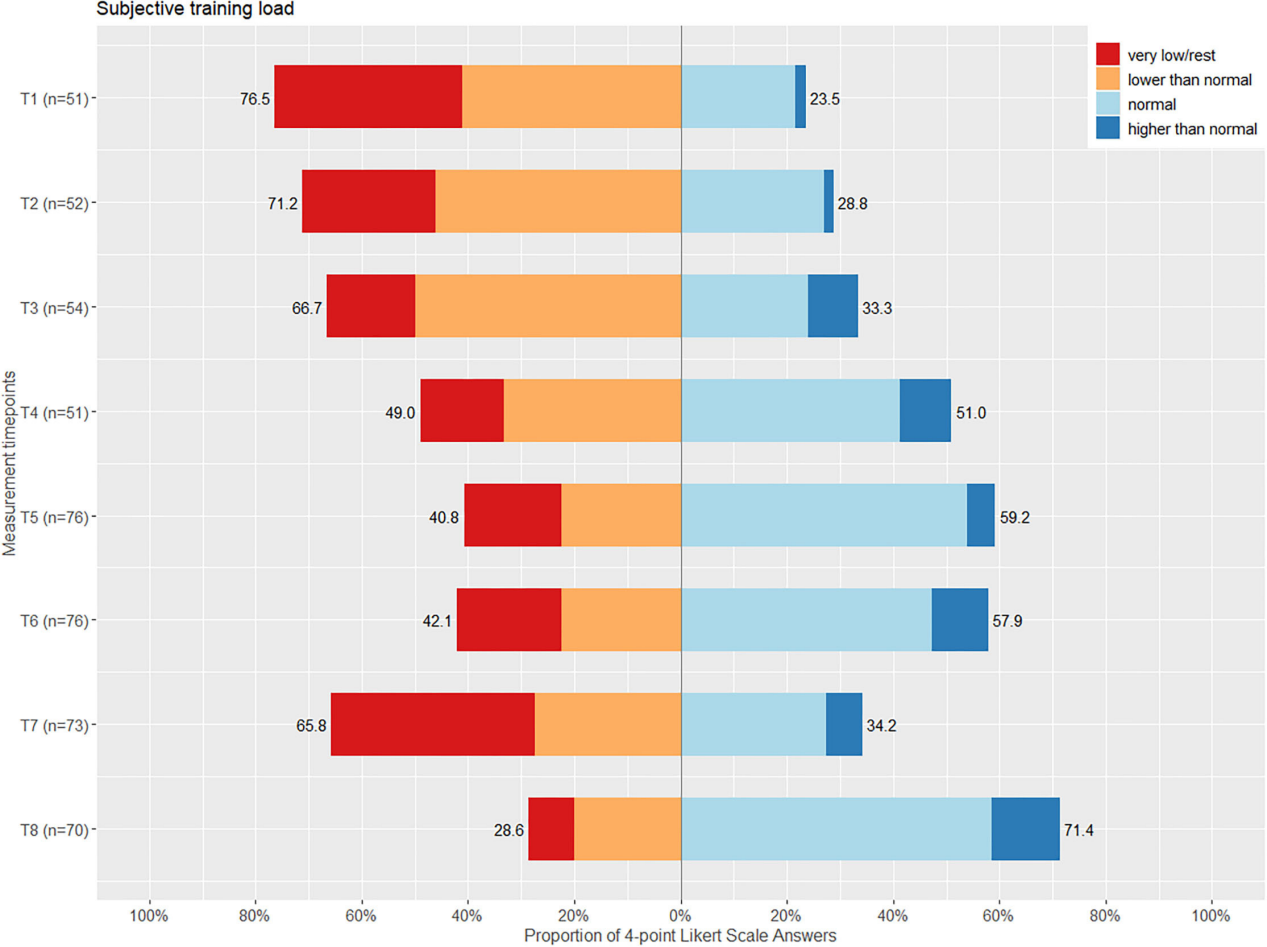
A bar chart of the 4-point Likert scale asking for the subjective training load in para-athletes, with the answers ranging from very low/rest to higher than normal with the according sum of percentages of positive or negative answers given per measurement time point (T1-8).

**Figure 2:**
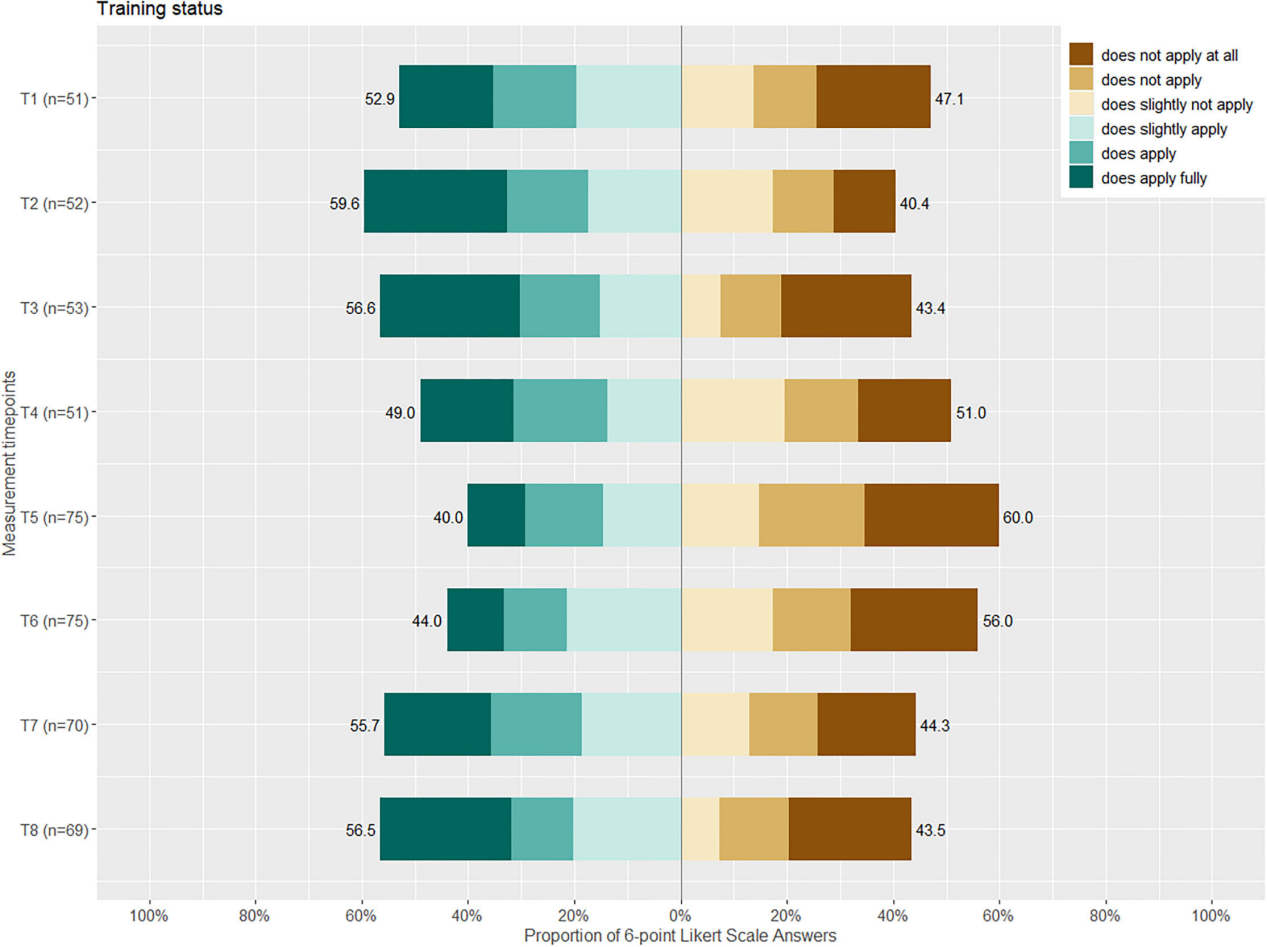
A bar chart of the 6-point Likert scale, given the statement to have been less active since the beginning of the COVID-19 pandemic, with the answers ranging from does not apply at all to does apply fully with the according sum of percentages of positive or negative answers given per measurement timepoint (T1-8).

Responses of the 4-point Likert scale on the perceived subjective training load and the training minutes per week can be found in Figure 1 and Table 2, respectively. An increase of the training minutes per week can be seen from the first assessment time point to the fourth, followed by a decrease until the seventh time point and the highest training minutes per week at the eighth measurement time point. The fluctuation of the training minutes per week is reflected with more positive or negative responses on the subjective training load, respectively (cf. Figure 1). No correlation was found between the subjective training load (*r*_*s*_= −0.06, *p* = 0.14) nor the training minutes per week (*r*_*s*_= −0.006, *p* = 0.87) and the PHQ-4 sum score in para-athletes. A weak positive correlation was found between the subjective training load (*r*_*s*_= 0.09, *p* = 0.02) and training minutes per week (*r*_*s*_=0.15, *p* = 0.0003) and the stress level in para-athletes. Responses of the general population sample to the statement to have been less active since the beginning of the COVID-19 pandemic were nearly equal over all measurement time points (cf. Figure 2). Contrary to the para-athlete sample, the matched general population sample showed a significant moderate positive correlation between the subjective activity status (*r*_*s*_= 0.031, *p* <0.0001) and the PHQ-4 sum score. Meaning the more the statement to be less active was denied, i.e., being more physically active, the lower PHQ-4 values were reported.

## Discussion

Mental health of para-athletes compared to a general population sample was investigated over the course of the COVID-19 pandemic from March 2020 to April 2021. Para-athletes had, on average, significantly lower PHQ-4 sum scores compared to the control group at all measurement time points; the hypothesis could not be confirmed. No relationship between subjective training load and training minutes per week with PHQ-4 values (depressive and anxiety symptoms) and the stress level could be found.

In the para-athlete cohort, the PHQ-4 sum scores at each time point ranged around a sum mean of 0.9–1.6. A cross-sectional study on mental health in able-bodied athletes in Australia during the lockdown from May to June 2020 reported higher PHQ-4 values, ranging from 2.5 to 3.8 (Facer-Childs et al., 2021). These sum scores are higher but still considered to be in a non-alarming range (Kroenke et al., 2009). Another study investigated the mental health status during lockdown, reopening and semi-lockdown in able-bodied elite athletes using the general health questionnaire-28. They found significantly higher scores of anxiety, insomnia, and depressive symptoms during the lockdown phase compared to the reopening and semi-lockdown and significantly higher depression scores during semi-lockdown compared to reopening (Mehrsafar et al., 2021). Slight changes in mental health, especially at the beginning of the semi-lockdown after the reopening phase, can also be found in the present study. Additionally, the highest value of the mean stress level was shown at the last measurement time point. This might be due to the time point, marking the final 6 months of preparation before the start of the postponed Tokyo 2020 Games. Para-athletes could be more stressed due to organization of training and pre-paralympic competitions. Changes of the mean level between the time points are reflected by some individuals reporting very high burden. On a group basis, the cohorts seem to be resilient; however, individuals can still be strongly affected by mental health stressors, emphasizing the individualized support need.

The presented significantly higher PHQ-4 sum scores in the general population compared to the para-athletes might give the impression that athletes can adapt better to stressful situations (Rubio et al., 2021). However, a study found no differences in resilience levels in athletes compared to non-athletes with easing restrictions after a COVID-19 lockdown (Knowles et al., 2021). Nonetheless, the lower PHQ-4 values in the para-athletes might be an anchoring effect. As para-athletes often suffered a major life-changing situation, e.g., severe trauma due to accident resulting in disability, the overall burden to elicit a significant impact on their mental health might need to be higher than in other persons (Denerel and Lima, 2022). A study comparing athletes with and without disabilities in Turkey showed higher scores in the depression anxiety stress scales-21 (DASS-21) in para-athletes assessed in summer 2021, reaching no statistical difference (Denerel and Lima, 2022). Investigations into the mental health status of US Olympic and Paralympic athletes showed significantly higher values in depression and anxiety scales in the paralympic cohort in a pre-COVID-19 assessment (Nabhan et al., 2021). Thus, leaving coping strategies highly individual and to be examined in further studies. An explanation for the general lower values of the PHQ-4 sum score in the paralympic cohort might be due to the additional support offered by the projects staff. If requested by the participant or when the PHQ-4 sum score of four was exceeded, a sport psychiatrist made personal contact to offer help if needed (Dallmann et al., 2021). This direct reaction or, simply, the knowledge of someone being available if urgently needed might be one factor of lower PHQ-4 values. Another explanation might be that, shortly after the postponement of the Tokyo 2020 Games, the financial support was reassured, or contracts extended. As only 24% of these athletes consider themselves as professionals, the focus and dependence of the sport might also be lower than in other elite athlete groups (Kubosch et al., 2021). The para-athletes may have had at least partly financial security and could further maintain their goal of participating in the paralympic games. The participants of the general population sample might have had more financial insecurities over the course of the pandemic reflected by greater PHQ-4 values.

The latent growth model showed no effect of sex or age on the mental health status of all the participants over all measurement time points. In contrast, higher resilience and less anxiety during the COVID-19 pandemic were found in male compared to female able-bodied athletes (Knowles et al., 2021). This is supported by a pre-COVID-19-conducted meta-analysis, finding female gender and younger age as determining factors in anxiety in elite athletes (Rice et al., 2019). A consensus statement on mental health of the IOC supports that female athletes may report two times as high depressive symptoms than male athletes (Reardon et al., 2019). Furthermore, another study on mental health during the COVID-19 pandemic showed slightly higher distress in mental health in females compared to males, reaching no significant difference (Denerel and Lima, 2022). The lack of age or sex as influencing factors in the mental health status in the present study might be due to the holistic influence of the pandemic or due to the relatively small sample size.

Training seemed to be reduced in the first measurement waves in the para-athletes. Fluctuations were small, but training load increased in general during the investigation time. Another study reported no impact of the pandemic on the training volume in para-cyclists and para-triathletes (Shaw et al., 2021). The participants of multiple countries (Canada, United Kingdom, USA, Australia, South Africa, and Belgium) were included in this study, making a direct comparison of restrictions and impact on the athletes difficult (Shaw et al., 2021). Furthermore, this para-athlete cohort reported to have difficulties organizing the training in the early phase of the pandemic (Kubosch et al., 2021). The present weak correlation between training load and the stress level might represent those difficulties, without having an impact on the PHQ-4 values. It could be discussed that, with postponement of the Tokyo 2020 Games and easing restrictions for professional sport to resume to normal training loads, factors influencing the mental health of the para-athletes were fast diminished. Contrarily, for the general population sample, a mild correlation of more inactivity and higher PHQ-4 values were found. A positive influence of physical activity on mental health has been shown in another study on mental health of the general population during COVID-19 (Petzold et al., 2020). Furthermore, these findings can be supported by the literature, considering physical activity as anxiolytic and antidepressant (Ensari et al., 2015).

This study comes with some limitations. First, due to the matching, not all measurement time points were the same in both samples. A greater general population sample could give more options to find the best match. However, a sample of above 8,000 participants represents already a large number of individuals and, as shown, gives in the majority of the cases a well-fitted match. Second, the para-athlete sample included participants from 16 years of age and the general population sample from 18 years. Therefore, no perfect age match for the 16-year-old para-athletes could be found. Nonetheless, especially in teenage ages, psychological status and demands can differ. A direct comparison would enhance the results of this study. Overall, some paired matches had an age difference with up to 7 years. Again, with greater samples of both cohorts, this might be compensated, and matching could be more precise. Nonetheless, the longitudinal monitoring gives great insights into the mental health status despite the smaller sample size. Furthermore, the duration of the participation of the projects varied between the two cohorts. Some participants of the para-athlete sample took part since more than a year, including weekly assessments of the mental health status. Constant monitoring could lead to both extrema: annoyance and sensitization. In contrast, the general population cohort was only asked to fill out the questionnaire at the described eight measurement time points. The self-reported measures needed to be relied upon. An in-depth interview could have yielded a more detailed outcome of the mental health status. To minimize influences of short-term participation, only the participants who took part in at least four assessment time points were included. A comparison to pre-pandemic data would contribute to classify the discovered differences and to highlight the actual effect of the COVID-19 pandemic. Furthermore, comparisons within the para-athlete group might give insights into influence of the category of sports (individual or team sports) on mental health and should be evaluated in future studies (Uroh and Adewunmi, 2021). Moreover, for confounding variables, e.g., dietary intake was not controlled for. And, last, both projects are correlative-observational studies. Therefore, no causal conclusions can be drawn from the data.

To conclude, the para-athlete cohort reported significantly lower PHQ-4 values compared to the general population sample, while no influence of sex and age was found. Factors of the lower PHQ-4 values of the paralympic cohort might be higher resilience due to anchoring effects or the additional support offered by the project staff and the fast security of sponsorships after the postponement of the Tokyo 2020 Games. For further estimation of the mental health in paralympic athletes during the pandemic, a comparison also to Olympic athletes might be reasonable. In addition, as the dynamic pandemic progresses, the mental health of para-athletes, athletes, and the general population should also be investigated to identify possible support needs.

## Data Availability Statement

The raw data supporting the conclusions of this article will be made available by the authors, without undue reservation.

## Ethics Statement

The studies involving human participants were reviewed and approved by Ethics Committee of the Albert-Ludwigs-University Freiburg; Hugstetter Str. 55, 79106 Freiburg, Germany and Ethics Committee of the Charité - Universitätsmedizin Berlin; Charitépl. 1, 10117 Berlin, Germany. The patients/participants provided their written informed consent to participate in this study.

## Author Contributions

ABu, EJK, and ABe contributed to conception and design of the study. VM and BB provided content to the database of one sample. ABe and MP provided content to the database of the other sample. ABu organized the joining of the databases and wrote the first draft of the manuscript. ABu and RL performed the statistical analysis. ABe and RL wrote sections of the manuscript. All authors contributed to manuscript revision, read, and approved the submitted version.

## Funding

The paralympic project has been funded by the Federal Ministry of the Interior, Building and Community of Germany (Grant No. ZMVI4-070404). We acknowledge support by the Open Access Publication Fund of the University of Freiburg. EJK is funded by the Berta-Ottenstein-Programme for Advanced Clinician Scientists, Faculty of Medicine, University of Freiburg.

## Conflict of Interest

The authors declare that the research was conducted in the absence of any commercial or financial relationships that could be construed as a potential conflict of interest.

## Publisher's Note

All claims expressed in this article are solely those of the authors and do not necessarily represent those of their affiliated organizations, or those of the publisher, the editors and the reviewers. Any product that may be evaluated in this article, or claim that may be made by its manufacturer, is not guaranteed or endorsed by the publisher.
