# Supplementary material for: Mental Health in German Paralympic Athletes During the 1st Year of the COVID-19 Pandemic Compared to a General Population Sample
Source: Front Sports Act Living. 2022 Apr 14;4:870692. doi: 10.3389/fspor.2022.870692 (PMC9047049; doi:10.3389/fspor.2022.870692)
Supplement: Supplementary file 1 [file Data_Sheet_1.docx]

Supplementary 1

Table S1. PHQ-4 sum score and stress level distribution in the para-athlete sample over each measurement timepoint (T1-8).

| Timepoint | PHQ-4 sum score in absolute numbers and percentage (%) | Stress level scale in absolute numbers and percentage (%) | Valid  N (%) | | Missing  N (%) |
| --- | --- | --- | --- | --- | --- |
| T1  (March 27 – April 6 2020) | \| 0 \| : \| 28 \| ( \| 54.9% \| ) \| \| --- \| --- \| --- \| --- \| --- \| --- \| \| 1 \| : \| 10 \| ( \| 19.6% \| ) \| \| 2 \| : \| 5 \| ( \| 9.8% \| ) \| \| 3 \| : \| 4 \| ( \| 7.8% \| ) \| \| 4 \| : \| 3 \| ( \| 5.9% \| ) \| \| 8 \| : \| 1 \| ( \| 2.0% \| ) \| | \| 0 \| : \| 9 \| ( \| 17.6% \| ) \| \| --- \| --- \| --- \| --- \| --- \| --- \| \| 1 \| : \| 6 \| ( \| 11.8% \| ) \| \| 2 \| : \| 10 \| ( \| 19.6% \| ) \| \| 3 \| : \| 11 \| ( \| 21.6% \| ) \| \| 4 \| : \| 4 \| ( \| 7.8% \| ) \| \| 5 \| : \| 4 \| ( \| 7.8% \| ) \| \| 6 \| : \| 3 \| ( \| 5.9% \| ) \| \| 7 \| : \| 2 \| ( \| 3.9% \| ) \| \| 8 \| : \| 1 \| ( \| 2.0% \| ) \| \| 9 \| : \| 1 \| ( \| 2.0% \| ) \| | 51 (65.4%) | | 27 (34.6%) |
| T2  (April 24 – May 4 2020) | \| 0 \| : \| 32 \| ( \| 61.5% \| ) \| \| --- \| --- \| --- \| --- \| --- \| --- \| \| 1 \| : \| 6 \| ( \| 11.5% \| ) \| \| 2 \| : \| 6 \| ( \| 11.5% \| ) \| \| 3 \| : \| 4 \| ( \| 7.7% \| ) \| \| 4 \| : \| 2 \| ( \| 3.8% \| ) \| \| 5 \| : \| 1 \| ( \| 1.9% \| ) \| \| 6 \| : \| 1 \| ( \| 1.9% \| ) \| | \| 0 \| : \| 8 \| ( \| 15.4% \| ) \| \| --- \| --- \| --- \| --- \| --- \| --- \| \| 1 \| : \| 8 \| ( \| 15.4% \| ) \| \| 2 \| : \| 7 \| ( \| 13.5% \| ) \| \| 3 \| : \| 6 \| ( \| 11.5% \| ) \| \| 4 \| : \| 8 \| ( \| 15.4% \| ) \| \| 5 \| : \| 7 \| ( \| 13.5% \| ) \| \| 6 \| : \| 3 \| ( \| 5.8% \| ) \| \| 7 \| : \| 3 \| ( \| 5.8% \| ) \| \| 8 \| : \| 2 \| ( \| 3.8% \| ) \| | 52 (66.7%) | | 26 (33.3%) |
| T3  (May 15 – May 25 2020) | \| 0 \| : \| 32 \| ( \| 59.3% \| ) \| \| --- \| --- \| --- \| --- \| --- \| --- \| \| 1 \| : \| 7 \| ( \| 13.0% \| ) \| \| 2 \| : \| 4 \| ( \| 7.4% \| ) \| \| 3 \| : \| 4 \| ( \| 7.4% \| ) \| \| 4 \| : \| 5 \| ( \| 9.3% \| ) \| \| 5 \| : \| 1 \| ( \| 1.9% \| ) \| \| 11 \| : \| 1 \| ( \| 1.9% \| ) \| | \| 0 \| : \| 10 \| ( \| 18.5% \| ) \| \| --- \| --- \| --- \| --- \| --- \| --- \| \| 1 \| : \| 7 \| ( \| 13.0% \| ) \| \| 2 \| : \| 10 \| ( \| 18.5% \| ) \| \| 3 \| : \| 7 \| ( \| 13.0% \| ) \| \| 4 \| : \| 3 \| ( \| 5.6% \| ) \| \| 5 \| : \| 7 \| ( \| 13.0% \| ) \| \| 6 \| : \| 4 \| ( \| 7.4% \| ) \| \| 7 \| : \| 1 \| ( \| 1.9% \| ) \| \| 8 \| : \| 4 \| ( \| 7.4% \| ) \| \| 10 \| : \| 1 \| ( \| 1.9% \| ) \| | 54 (69.2%) | | 24 (30.8%) |
| T4  (June 5 – June 15 2020) | \| 0 \| : \| 33 \| ( \| 64.7% \| ) \| \| --- \| --- \| --- \| --- \| --- \| --- \| \| 1 \| : \| 8 \| ( \| 15.7% \| ) \| \| 2 \| : \| 3 \| ( \| 5.9% \| ) \| \| 3 \| : \| 1 \| ( \| 2.0% \| ) \| \| 4 \| : \| 3 \| ( \| 5.9% \| ) \| \| 5 \| : \| 2 \| ( \| 3.9% \| ) \| \| 6 \| : \| 1 \| ( \| 2.0% \| ) \| | \| 0 \| : \| 7 \| ( \| 13.7% \| ) \| \| --- \| --- \| --- \| --- \| --- \| --- \| \| 1 \| : \| 6 \| ( \| 11.8% \| ) \| \| 2 \| : \| 11 \| ( \| 21.6% \| ) \| \| 3 \| : \| 8 \| ( \| 15.7% \| ) \| \| 4 \| : \| 4 \| ( \| 7.8% \| ) \| \| 5 \| : \| 4 \| ( \| 7.8% \| ) \| \| 6 \| : \| 5 \| ( \| 9.8% \| ) \| \| 7 \| : \| 4 \| ( \| 7.8% \| ) \| \| 8 \| : \| 2 \| ( \| 3.9% \| ) \| | 51 (65.4%) | | 27 (34.6%) |
| T5  (September 25 – October 5, 2020) | \| 0 \| : \| 44 \| ( \| 57.9% \| ) \| \| --- \| --- \| --- \| --- \| --- \| --- \| \| 1 \| : \| 10 \| ( \| 13.2% \| ) \| \| 2 \| : \| 10 \| ( \| 13.2% \| ) \| \| 3 \| : \| 3 \| ( \| 3.9% \| ) \| \| 4 \| : \| 4 \| ( \| 5.3% \| ) \| \| 5 \| : \| 1 \| ( \| 1.3% \| ) \| \| 6 \| : \| 2 \| ( \| 2.6% \| ) \| \| 8 \| : \| 2 \| ( \| 2.6% \| ) \| | \| 0 \| : \| 8 \| ( \| 10.5% \| ) \| \| --- \| --- \| --- \| --- \| --- \| --- \| \| 1 \| : \| 7 \| ( \| 9.2% \| ) \| \| 2 \| : \| 11 \| ( \| 14.5% \| ) \| \| 3 \| : \| 10 \| ( \| 13.2% \| ) \| \| 4 \| : \| 12 \| ( \| 15.8% \| ) \| \| 5 \| : \| 12 \| ( \| 15.8% \| ) \| \| 6 \| : \| 8 \| ( \| 10.5% \| ) \| \| 7 \| : \| 4 \| ( \| 5.3% \| ) \| \| 8 \| : \| 3 \| ( \| 3.9% \| ) \| \| 9 \| : \| 1 \| ( \| 1.3% \| ) \| | 76 (97.4%) | | 2 (2.6%) |
| T6  (October 23 – November 2 2020) | \| 0 \| : \| 46 \| ( \| 60.5% \| ) \| \| --- \| --- \| --- \| --- \| --- \| --- \| \| 1 \| : \| 3 \| ( \| 3.9% \| ) \| \| 2 \| : \| 5 \| ( \| 6.6% \| ) \| \| 3 \| : \| 8 \| ( \| 10.5% \| ) \| \| 4 \| : \| 8 \| ( \| 10.5% \| ) \| \| 5 \| : \| 2 \| ( \| 2.6% \| ) \| \| 7 \| : \| 1 \| ( \| 1.3% \| ) \| \| 8 \| : \| 1 \| ( \| 1.3% \| ) \| \| 12 \| : \| 2 \| ( \| 2.6% \| ) \| | \| 0 \| : \| 9 \| ( \| 11.8% \| ) \| \| --- \| --- \| --- \| --- \| --- \| --- \| \| 1 \| : \| 2 \| ( \| 2.6% \| ) \| \| 2 \| : \| 19 \| ( \| 25.0% \| ) \| \| 3 \| : \| 9 \| ( \| 11.8% \| ) \| \| 4 \| : \| 8 \| ( \| 10.5% \| ) \| \| 5 \| : \| 11 \| ( \| 14.5% \| ) \| \| 6 \| : \| 7 \| ( \| 9.2% \| ) \| \| 7 \| : \| 4 \| ( \| 5.3% \| ) \| \| 8 \| : \| 6 \| ( \| 7.9% \| ) \| \| 9 \| : \| 1 \| ( \| 1.3% \| ) \| | 76 (97.4%) | | 2 (2.6%) |
| T7  (January 1 – January 11 2021) | \| 0 \| : \| 46 \| ( \| 63.0% \| ) \| \| --- \| --- \| --- \| --- \| --- \| --- \| \| 1 \| : \| 8 \| ( \| 11.0% \| ) \| \| 2 \| : \| 7 \| ( \| 9.6% \| ) \| \| 3 \| : \| 2 \| ( \| 2.7% \| ) \| \| 4 \| : \| 6 \| ( \| 8.2% \| ) \| \| 5 \| : \| 1 \| ( \| 1.4% \| ) \| \| 6 \| : \| 1 \| ( \| 1.4% \| ) \| \| 8 \| : \| 1 \| ( \| 1.4% \| ) \| \| 12 \| : \| 1 \| ( \| 1.4% \| ) \| | \| 0 \| : \| 17 \| ( \| 23.3% \| ) \| \| --- \| --- \| --- \| --- \| --- \| --- \| \| 1 \| : \| 5 \| ( \| 6.8% \| ) \| \| 2 \| : \| 12 \| ( \| 16.4% \| ) \| \| 3 \| : \| 16 \| ( \| 21.9% \| ) \| \| 4 \| : \| 11 \| ( \| 15.1% \| ) \| \| 5 \| : \| 6 \| ( \| 8.2% \| ) \| \| 7 \| : \| 4 \| ( \| 5.5% \| ) \| \| 8 \| : \| 1 \| ( \| 1.4% \| ) \| \| 9 \| : \| 1 \| ( \| 1.4% \| ) \| | 73 (93.6%) | 5 (6.4%) | |
| T8  (March 26 to April 5 2021) | \| 0 \| : \| 41 \| ( \| 58.6% \| ) \| \| --- \| --- \| --- \| --- \| --- \| --- \| \| 1 \| : \| 7 \| ( \| 10.0% \| ) \| \| 2 \| : \| 5 \| ( \| 7.1% \| ) \| \| 3 \| : \| 6 \| ( \| 8.6% \| ) \| \| 4 \| : \| 7 \| ( \| 10.0% \| ) \| \| 6 \| : \| 1 \| ( \| 1.4% \| ) \| \| 7 \| : \| 1 \| ( \| 1.4% \| ) \| \| 8 \| : \| 1 \| ( \| 1.4% \| ) \| \| 9 \| : \| 1 \| ( \| 1.4% \| ) \| | \| 0 \| : \| 7 \| ( \| 10.0% \| ) \| \| --- \| --- \| --- \| --- \| --- \| --- \| \| 1 \| : \| 4 \| ( \| 5.7% \| ) \| \| 2 \| : \| 10 \| ( \| 14.3% \| ) \| \| 3 \| : \| 15 \| ( \| 21.4% \| ) \| \| 4 \| : \| 9 \| ( \| 12.9% \| ) \| \| 5 \| : \| 7 \| ( \| 10.0% \| ) \| \| 6 \| : \| 6 \| ( \| 8.6% \| ) \| \| 7 \| : \| 5 \| ( \| 7.1% \| ) \| \| 8 \| : \| 4 \| ( \| 5.7% \| ) \| \| 9 \| : \| 3 \| ( \| 4.3% \| ) \| | 70 (89.7%) | 8 (10.3%) | |

Table S2. PHQ-4 sum score distribution in the general population sample over each measurement timepoint (T1-8).

| Timepoint | PHQ-4 sum score in absolute numbers and percentage (%) | Valid  N (%) | Missing  N (%) |
| --- | --- | --- | --- |
| T1  (March 27 – April 6 2020) | \| 0 \| : \| 6 \| ( \| 11.8% \| ) \| \| --- \| --- \| --- \| --- \| --- \| --- \| \| 1 \| : \| 8 \| ( \| 15.7% \| ) \| \| 2 \| : \| 8 \| ( \| 15.7% \| ) \| \| 3 \| : \| 8 \| ( \| 15.7% \| ) \| \| 4 \| : \| 2 \| ( \| 3.9% \| ) \| \| 5 \| : \| 6 \| ( \| 11.8% \| ) \| \| 6 \| : \| 4 \| ( \| 7.8% \| ) \| \| 7 \| : \| 1 \| ( \| 2% \| ) \| \| 8 \| : \| 3 \| ( \| 5.9% \| ) \| \| 9 \| : \| 2 \| ( \| 3.9% \| ) \| \| 10 \| : \| 2 \| ( \| 3.9% \| ) \| \| 11 \| : \| 1 \| ( \| 2% \| ) \| | 51 (65.4%) | 27 (34.6%) |
| T2  (April 24 – May 4 2020) | \| 0 \| : \| 4 \| ( \| 7.7% \| ) \| \| --- \| --- \| --- \| --- \| --- \| --- \| \| 1 \| : \| 6 \| ( \| 11.5% \| ) \| \| 2 \| : \| 10 \| ( \| 19.2% \| ) \| \| 3 \| : \| 8 \| ( \| 15.4% \| ) \| \| 4 \| : \| 8 \| ( \| 15.4% \| ) \| \| 5 \| : \| 6 \| ( \| 11.5% \| ) \| \| 6 \| : \| 1 \| ( \| 2% \| ) \| \| 8 \| : \| 3 \| ( \| 5.7% \| ) \| \| 9 \| : \| 3 \| ( \| 5.7% \| ) \| \| 10 \| : \| 2 \| ( \| 3.9% \| ) \| \| 12 \| : \| 1 \| ( \| 2% \| ) \| | 52 (66.7%) | 26 (33.3%) |
| T3  (May 15 – May 25 2020) | \| 0 \| : \| 5 \| ( \| 9.4% \| ) \| \| --- \| --- \| --- \| --- \| --- \| --- \| \| 1 \| : \| 6 \| ( \| 11.3% \| ) \| \| 2 \| : \| 8 \| ( \| 15% \| ) \| \| 3 \| : \| 10 \| ( \| 18.8% \| ) \| \| 4 \| : \| 5 \| ( \| 9.4% \| ) \| \| 5 \| : \| 4 \| ( \| 7.5% \| ) \| \| 6 \| : \| 2 \| ( \| 3.8% \| ) \| \| 7 \| : \| 5 \| ( \| 9.4% \| ) \| \| 8 \| : \| 4 \| ( \| 7.5% \| ) \| \| 9 \| : \| 2 \| ( \| 3.7% \| ) \| \| 11 \| : \| 1 \| ( \| 1.9% \| ) \| \| 12 \| : \| 1 \| ( \| 1.9% \| ) \| | 53 (67.9%) | 25 (32.1%) |
| T4  (June 5 – June 15 2020) | \| 0 \| : \| 9 \| ( \| 17.6% \| ) \| \| --- \| --- \| --- \| --- \| --- \| --- \| \| 1 \| : \| 9 \| ( \| 17.6% \| ) \| \| 2 \| : \| 5 \| ( \| 9.8% \| ) \| \| 3 \| : \| 9 \| ( \| 17.6% \| ) \| \| 4 \| : \| 4 \| ( \| 7.8% \| ) \| \| 5 \| : \| 3 \| ( \| 5.8% \| ) \| \| 6 \| : \| 3 \| ( \| 5.8% \| ) \| \| 7 \| : \| 3 \| ( \| 5.8% \| ) \| \| 8 \| : \| 1 \| ( \| 2% \| ) \| \| 9 \| : \| 3 \| ( \| 5.9% \| ) \| \| 10 \| : \| 1 \| ( \| 2% \| ) \| \| 12 \| : \| 1 \| ( \| 2% \| ) \| | 51 (65.4%) | 27 (34.6%) |
| T5  (September 25 – October 5, 2020) | \| 0 \| : \| 15 \| ( \| 20% \| ) \| \| --- \| --- \| --- \| --- \| --- \| --- \| \| 1 \| : \| 9 \| ( \| 12% \| ) \| \| 2 \| : \| 13 \| ( \| 17.3% \| ) \| \| 3 \| : \| 6 \| ( \| 8% \| ) \| \| 4 \| : \| 6 \| ( \| 8% \| ) \| \| 5 \| : \| 5 \| ( \| 6.7% \| ) \| \| 6 \| : \| 4 \| ( \| 5.3% \| ) \| \| 8 \| : \| 8 \| ( \| 10.6% \| ) \| \| 9 \| : \| 3 \| ( \| 4% \| ) \| \| 10 \| : \| 1 \| ( \| 1.3% \| ) \| \| 11 \| : \| 2 \| ( \| 2.7% \| ) \| \| 12 \| : \| 3 \| ( \| 4% \| ) \| | 75 (96.2%) | 3 (3.8%) |
| T6  (October 23 – November 2 2020) | \| 0 \| : \| 12 \| ( \| 16% \| ) \| \| --- \| --- \| --- \| --- \| --- \| --- \| \| 1 \| : \| 12 \| ( \| 16% \| ) \| \| 2 \| : \| 10 \| ( \| 13.3% \| ) \| \| 3 \| : \| 9 \| ( \| 12% \| ) \| \| 4 \| : \| 11 \| ( \| 14.6% \| ) \| \| 5 \| : \| 5 \| ( \| 6.6% \| ) \| \| 6 \| : \| 3 \| ( \| 4% \| ) \| \| 7 \| : \| 3 \| ( \| 4% \| ) \| \| 8 \| : \| 3 \| ( \| 4% \| ) \| \| 9 \| : \| 2 \| ( \| 2.7% \| ) \| \| 10 \| : \| 1 \| ( \| 1.3% \| ) \| \| 11 \| : \| 1 \| ( \| 1.3% \| ) \| \| 12 \| : \| 3 \| ( \| 4% \| ) \| | 75 (96.2%) | 3 (3.8%) |
| T7  (January 1 – January 11 2021) | \| 0 \| : \| 11 \| ( \| 16.2% \| ) \| \| --- \| --- \| --- \| --- \| --- \| --- \| \| 1 \| : \| 7 \| ( \| 10.3% \| ) \| \| 2 \| : \| 12 \| ( \| 17.6% \| ) \| \| 3 \| : \| 4 \| ( \| 5.9% \| ) \| \| 4 \| : \| 8 \| ( \| 11.8% \| ) \| \| 5 \| : \| 8 \| ( \| 11.8% \| ) \| \| 6 \| : \| 4 \| ( \| 5.9% \| ) \| \| 7 \| : \| 3 \| ( \| 4.4% \| ) \| \| 8 \| : \| 5 \| ( \| 7.3% \| ) \| \| 9 \| : \| 2 \| ( \| 3% \| ) \| \| 10 \| : \| 2 \| ( \| 3% \| ) \| \| 11 \| : \| 1 \| ( \| 1.5% \| ) \| \| 12 \| : \| 1 \| ( \| 1.5% \| ) \| | 68 (87.2%) | 10 (12.8%) |
| T8  (March 26 to April 5 2021) | \| 0 \| : \| 8 \| ( \| 11.6% \| ) \| \| --- \| --- \| --- \| --- \| --- \| --- \| \| 1 \| : \| 11 \| ( \| 15.9% \| ) \| \| 2 \| : \| 10 \| ( \| 14.5% \| ) \| \| 3 \| : \| 7 \| ( \| 10.1% \| ) \| \| 4 \| : \| 7 \| ( \| 10.1% \| ) \| \| 5 \| : \| 7 \| ( \| 10.1% \| ) \| \| 6 \| : \| 5 \| ( \| 7.3% \| ) \| \| 7 \| : \| 2 \| ( \| 2.9% \| ) \| \| 8 \| : \| 4 \| ( \| 5.8% \| ) \| \| 10 \| : \| 5 \| ( \| 7.3% \| ) \| \| 11 \| : \| 1 \| ( \| 1.5% \| ) \| \| 12 \| : \| 2 \| ( \| 2.9% \| ) \| | 69 (88.5%) | 9 (11.5%) |
